# Performance of Spot Photoscreener in Detecting Amblyopia Risk Factors in Chinese Pre-school and School Age Children Attending an Eye Clinic

**DOI:** 10.1371/journal.pone.0149561

**Published:** 2016-02-16

**Authors:** Yajun Mu, Hua Bi, Edgar Ekure, Gang Ding, Nan Wei, Ning Hua, Xuehan Qian, Xiaorong Li

**Affiliations:** 1 Department of strabismus and pediatric ophthalmology, Tianjin Medical University Eye Hospital, Tianjin, China; 2 College of Optometry, Nova Southeastern University, Fort Lauderdale, Florida, United States of America; 3 Department of retina and vitreous, Tianjin Medical University Eye Hospital, Tianjin, China; Shenzhen Institutes of Advanced Technology, CHINA

## Abstract

**Purpose:**

To evaluate the effectiveness of Spot photoscreener in detecting amblyopia risk factors meeting 2013 the American Association of Pediatric Ophthalmology and Strabismus (AAPOS) criteria in Chinese preschool and school-age children.

**Methods:**

One hundred and fifty-five children (310 eyes), aged between 4 to 7 years (5.74 ± 1.2 years) underwent complete ophthalmologic examination, photoscreening, and cycloplegic retinoscopy refraction. The agreement of the results obtained with the photoscreening and retinoscopy was evaluated by linear regression and Bland-Altman plots. The sensitivity and specificity of detecting amblyopia risk factors were calculated based on the AAPOS 2013 guidelines. The overall effectiveness of detecting amblyopia risk factors was analyzed with Receiver Operating Characteristic (ROC) curves.

**Result:**

The mean refractive errors measured with the Spot were: spherical equivalent (SE) = 0.70 ± 1.99 D, J0 = 0.87 ± 1.01 D, J45 = 0.09 ± 0.60 D. The mean results from retinoscopy were: SE = 1.19 ± 2.22 D, J0 = 0.77 ± 1.00 D, J45 = -0.02 ± 0.45 D. There was a strong linear agreement between results obtained from those two methods (R^2^ = 0.88, P<0.01). Bland–Altman plot indicated a moderate agreement of cylinder values between the two methods. Based on the criteria specified by the AAPOS 2013 guidelines, the sensitivity and specificity (in respective order) for detecting hyperopia were 98.31% and 97.14%; for detecting myopia were 78.50% and 88.64%; for detecting astigmatism were 90.91% and 80.37%; for detecting anisometropia were 93.10% and 85.25%; and for detection of strabismus was 77.55% and 88.18%.

**Conclusion:**

The refractive values measured from Spot photoscreener showed a moderate agreement with the results from cycloplegic retinoscopy refraction, however there was an overall myopic shift of -0.49D. The performance in detecting individual amblyopia risk factors was satisfactory, but could be further improved by optimizing criteria based on ROC curves.

## Introduction

Amblyopia is the most common cause of preventable vision impairment in children with a prevalence of 1.6% to 3.6% in industrialized nations[1]. Amblyopia often leads to a decrease in the quality of life and potential bilateral impairment in visual function if left untreated[2]. The risk factors for amblyopia include strabismus, ametropia, and optical deprivation[3]. Amblyopia can be treated successfully within the critical developmental period by prescribing glasses and/or occlusion therapy of the non-amblyopic eye[1]. The US Preventive Services Task Force (USPSTF) statement indicates that detection and treatment of amblyopia in children between the ages of 3 to 5 years leads to great improvement of visual acuity[4]. However, there is insufficient evidence to assess the benefits and/or harms of vision screening for children younger than 3 years old[4]. The Pediatric Eye Disease Investigator Group considers that the optimal time for the treatment amblyopia is when the children are younger than 7 years old[5]. Since amblyopia is preventable and treatable for early stage, vision screening is widely recommended by the American Association of Pediatric Ophthalmology and Strabismus (AAPOS), the American Academy of Pediatrics (AAP), the US Preventive Services Task Force (USPSTF)[4, 6]. Two historical comparative studies have associated the introduction of vision screening with a reduction of the prevalence of amblyopia[7, 8].

Instrument-based screening is a quick process that needs little cooperation of children and has been regarded as the preferred option for vision screening especially for developmentally delayed and preverbal children [4, 9]. Spot is a newly developed portable hand-held infrared photoscreener (Welch Allyn, Skaneateles Falls, NY). During the photoscreening, the examiner asks the subject to look at the instrument binocularly. With the image of the red reflex successfully acquired, the device automatically calculates the noncycloplegic refractive status, pupil size, interpupilary distance, and gaze deviation immediately. The device will flag a referral for complete eye examination if significant refractive error, anisometropia, anisocoria or strabismus are detected [10, 11]. The Spot shows great promise in large scale screening for the following reasons. First, it is performed approximately one meter from the children. This working distance can keep the children relaxed and is convenient for those with disabilities such as polimyelities and autism. Second, the data acquisition time is short, within 2 seconds, since both eyes are exanimated simultaneously [11]. This is significant in China considering its large population. Third, it is automated and can be used by lay screeners. This is particularly important in China where there are very few professional eyecare specialists. Fourth, the device is battery-operated and portable and therefore, can be used in various screening environments [10].

Recently, there have been some reports of the sensitivity and specificity of the Spot for screening amblyopia risk factors[10–12]. Although these studies reported reasonably high sensitivity and specificity (sensitivity ranges from87% to 89%, specificity ranges from 71% to 75.9%) for detecting amblyopia risk factors based on the criteria defined by AAPOS guidelines[13] or the manufacturer, whether the criteria can be optimized to achieve its best performance is unknown. Besides, all those studies were done in Caucasian populations. The performance of the Spot in the detection of amblyopia risk factors has not been determined for the Chinese populations, which has a high prevalence of amblyopia, mainly caused by refractive errors and strabismus[14]. Therefore, the purpose of the present study was to evaluate the effectiveness of Spot in detecting amblyopia risk factors using AAPOS 2013 guidelines in Chinese pre-school and school age children and to determine how it could be improved by optimizing the guidelines for detecting amblyopia risk factors based on ROC analysis.

## Method

The ethics Board of Tianjin Medical University Eye Hospital approved the study and parental consent was obtained prior to the start of the study. All questions and concerns were addressed before the consent forms were signed. The conduct of the study followed the tenets of the Declaration of Helsinki. Considering the low prevalence of optical deprivation such as congenital cataract or ptosis, those diseases were excluded in our study. Children attending the eye hospital either for screening or for a check-up within the age group of 4 to 7 years were recruited in this study.

All patients were examined in the following order: (1) complete ophthalmologic examination; (2) photoscreening with Spot; (3) cycloplegia; (4) retinoscopy. In the photoscreening procedure, the Spot was performed at a distance of one meter from the child. The Spot measurement range is +/- 7.50 D. If the refraction was out of range, >7.50 or <-7.50 would be displayed on the touch screen[15]. The test was conducted by nonspecialist trained staff who attempted to obtain results from each child in three trials or less. Tropicamide and Phenylephrine eye drops were instilled into each eye every five minutes for 20 minutes and retinoscopy was performed 20 to 25 minutes following the final instillation. Retinoscopy was done by an expert optometrist. Best Corrected Visual Acuity (BCVA) was recorded in either eye. The optometrist was masked from results of the Spot photoscreener to avoid potential bias. Strabismus was detected by an ophthalmologist using the cover-uncover test. If manifest strabismus was found, the amount of deviation was determined by the prism nulling method.

Refractive errors (Spherical [S], Cylinder [C], axis [a]) were measured five times by retinoscopy in each eye, and mean vector value was calculated as final result. Spherical equivalent (SE) and vector presentation of astigmatism J0 and J45 were calculated according to the following formulas: *SE* = *S* + *C* / 2; *J*0 = (−*C* / 2)*cos(2 * ∂); *J*45 = (−*C* / 2)*sin(2 * ∂). Anisometropia was calculated as the interocular difference in SE.

Amblyopia risk factors were based on the AAPOS 2013 guidelines for ages over 48 months: hyperopia >3.5 D in any meridian, myopia >-1.5 D in any meridian, astigmatism >1.5 D in any meridian; anisometropia >1.5D, manifest strabismus in primary position >8PD [13]. Both Spot and retinoscopy were analyzed based on these criteria. Amblyopia was defined based on the Preferred Practice Pattern (PPP) [2]: unilateral amblyopia > = 2 line interocular difference; bilateral amblyopia: ages > = 4 years: visual acuity worse than 20/40 in either eye.

Descriptive statistics included measurements of means, standard deviations and frequencies. Since Kolmogorov-Smirnov test indicated that data were not normally distributed, Wilcoxon signed-rank test was applied to test if the difference between the results obtained from Spot photoscreener and retinoscopy was significant. Linear, quadratic, cubic models were constructed to assess the correlation between the results obtained from those two methods. Bland-Altman plots were used to assess the agreement between Spot and retinoscopy. Receiver Operating Characteristic (ROC) curve was employed to select the best cutoff points related to appropriate sensitivity and specificity of the Spot to detect amblyopia risk factors. All statistical analyses were performed using SPSS statistical package 19 (SPSS, IBM, Chicago, IL, USA). Statistical significance was defined as p<0.05.

## Results

**Fig 1:**
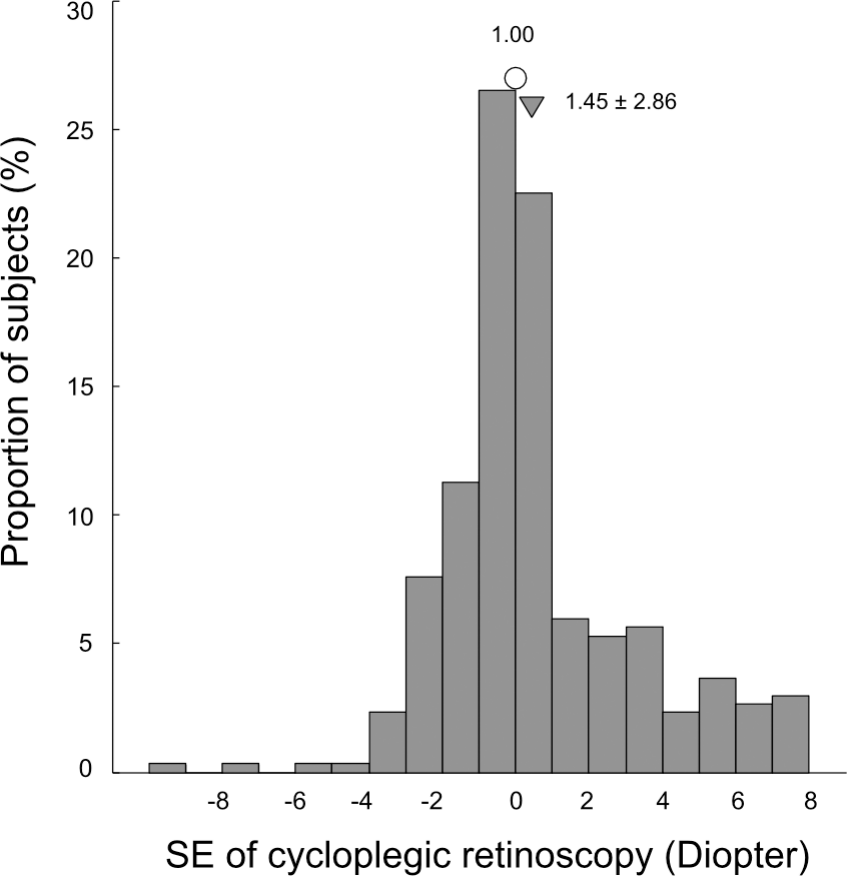
Histogram illustrating the distribution of spherical equivalent.

A total of 168 children were screened. Measurement could not be done in 13 (8.4%) children. Among those 13 children, five would not cooperate due to fear, three had high hyperopia accompanied with esotropia, two had congenital ptosis, two had congenital nystagmus, and one had congenital cataract. Measurement was successfully obtained from155 children (310 eyes), with age ranging from 4–7 years (means 5.74 ± 1.20 years). Seventy-one (45.8%) were girls and 84 (54.2%) were boys. Twenty-six children (16.8%) had amblyopia as defined by guidelines of PPP [2]. One hundred and fifteen (74.2%) children had amblyopia risk factors as defined by 2013 AAPOS criteria according to the result of cycloplegic retinoscopy. Of these, 65 children had hyperopia, 28 children had myopia, 59 children had astigmatism, 32 children had anisometropia, and 37 children had strabismus. The distribution of spherical equivalent is shown in Fig 1.

The mean refractive errors measured with Spot photoscreener and cycloplegic retinoscopy are summarized in Table 1. There were significant differences of SEs and cylinder power (J0 and J45) between the results obtained from the two methods (P < 0.01, Wilcoxon signed-rank test).

**Table 1:** The mean spherical equivalent (SE) and Jackson cross cylinder power values obtained with the Spot and cycloplegic retinoscopy.

	SE (D)	J0 (D)	J45 (D)
Spot	0.70±1.99	0.87±1.01	0.09±0.60
Retinoscopy	1.19±2.22	0.77±1.00	-0.02±0.45
	P<0.01	P<0.01	P<0.01

**Fig 2:**
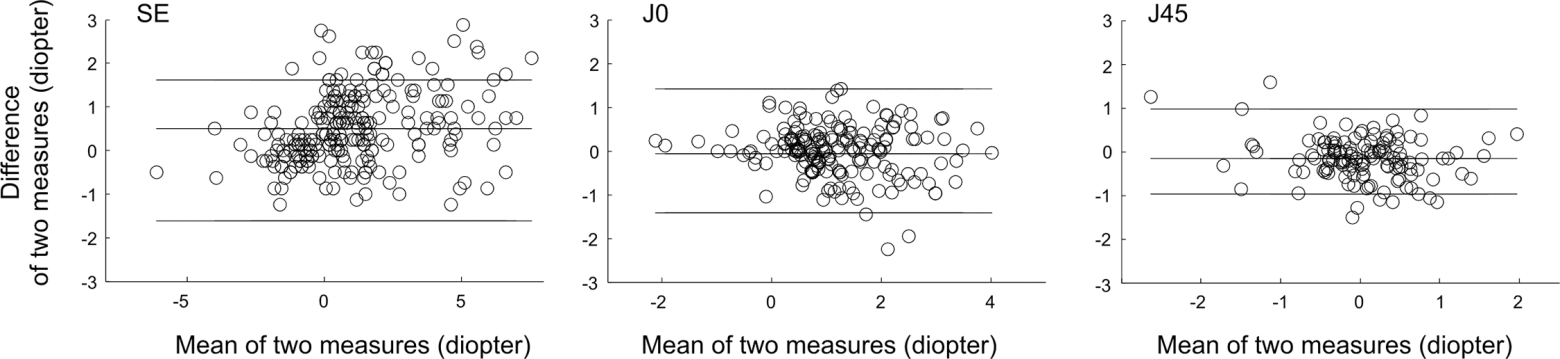
Bland-Altman plots showing agreement between the Spot and cycloplegic retinoscopy (CR) for SE, J0 and J45 values.

The mean difference of refractive errors between Spot photoscreener and cycloplegic retinoscopy are showed in Table 2. The difference (SSE -CRSE) is plotted against the average values [(CRSE + SSE)/ 2] in Fig 2A. In 76.3% of the subjects, the differences (SSE—CRSE) were within ± 1.0 D. Meanwhile, the differences of J0 in 92.7% of the subjects and the differences of J45 values in of 93.4% of the subjects were within ±1.0 D (Fig 2B and 2C).

**Table 2:** The difference of spherical equivalent (SE) and Jackson cross cylinder power values obtained with the Spot and cycloplegic retinoscopy.

	SE (D)	J0 (D)	J45 (D)
Mean ± SD	-0.49 ± 0.78	0.10 ± 0.65	0.11 ± 0.47
95% LOA	-2.96 to +1.06	-1.20 to +1.40	-0.83 to +1.05

**Fig 3:**
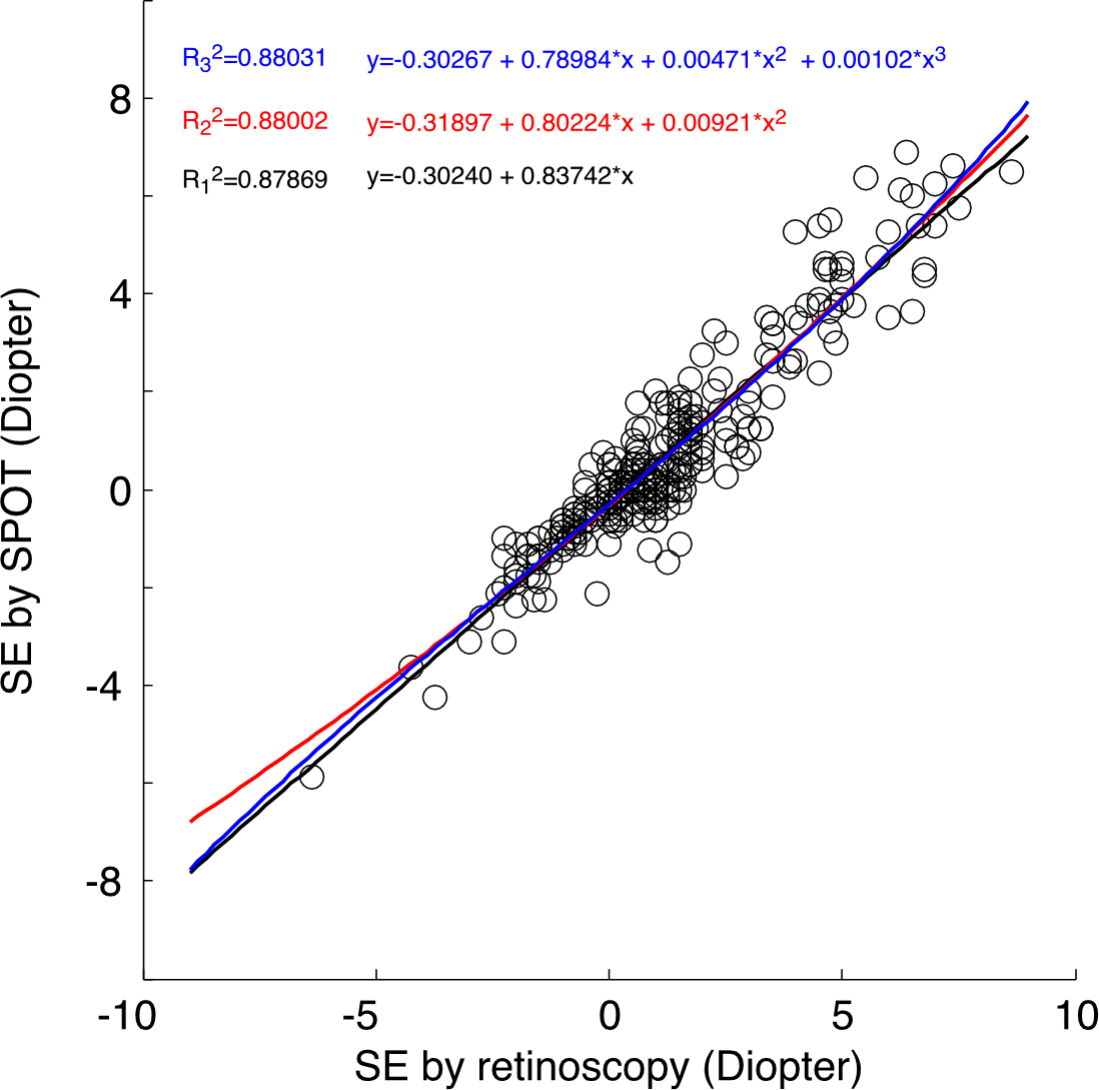
The correlation between SE measured by retinoscopy and Spot.

Regression was used to evaluate the quantitative relationship between the results of the Spot and cycloplegic retinoscopy. For spherical equivalent, a linear regression model, (*SEspot* = −0.30 + 0.84 × *SEcr*, R^2^ = 0.88, P<0.01, black line Fig 3) captured a majority of the variance and indicated strong linear correlation. Quadratic and cubic models did not improve the explained variations much, with R^2^ = 0.80 for both quadratic and cubic fitting (red and blue lines in Fig 3).

For J0, the linear correlation between retinoscopy and Spot was not as strong (*SEspot* = 0.26 + 0.80 × *SEcr*, R^2^ = 0.63, P <0.01). Quadratic and cubic fitting did not improve R^2^ much (R^2^ = 0.64 for quadratic and cubic fitting). For J45, the correlation between retinoscopy and Spot was further reduced (R^2^ = 0.24 for linear fitting R^2^ = 0.64 for both quadratic and cubic fitting). This may be due to the fact that most subjects had low astigmatism.

The sensitivity and specificity of the Spot in detecting amblyopia risk factors according to the AAPOS criteria are shown in Table 3.

**Table 3:** Sensitivity and specificity of identifying amblyopia risk factors with AAPOS criteria.

AAPOS criteria	Hyperopia	Myopia	Astigmatism	Anisometropia	Strabismus
	>3.5D	>-1.5D	>1.5D	>1.5D	>8PD
Sensitivity	79.66%	85.51%	84.09%	75.86%	85.00%
Specificity	99.43%	79.55%	86.92%	95.08%	77.50%

**Fig 4:**
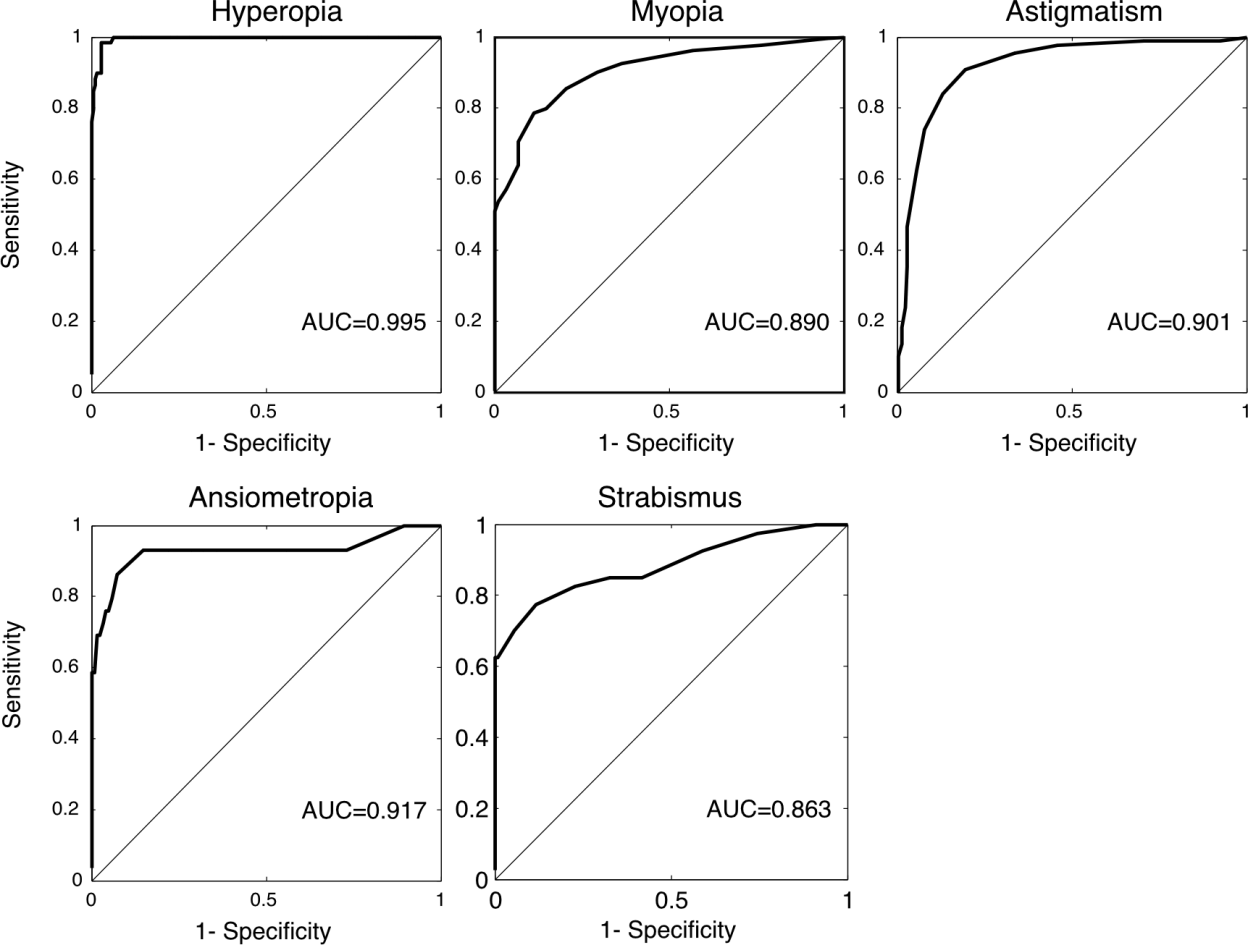
ROC curves for detecting the risk factors of amblyopia (hyperopia, myopia, astigmatism, anisometropia, and strabismus).

The ROC curve was used to determine the effectiveness of the Spot in detecting amblyopia risk factors (Fig 4). The optimal cutoff and sensitivity and specificity are shown in Table 4. Overall, 115 patients (74.2%) had amblyopia risk factors with a sensitivity of 94.79% and specificity of 85% for Spot in detecting the amblyopia risk factors.

**Table 4:** Sensitivity and specificity to detect AAPOS 2013 defined amblyopia risk factors with Spot cutoff values derived from ROC curves.

Optimized criteria	Hyperopia	Myopia	Astigmatism	Anisometropia	Strabismus
	>2.375D	>-2.00D	>1.25D	>1.125D	>12PD
Sensitivity	98.31%	78.50%	90.91%	93.10%	77.55%
Specificity	97.14%	88.64%	80.37%	85.25%	88.18%

## Discussion

In this study we compared the refractive error estimates of Spot to the gold-standard cycloplegic retinoscopy and evaluated its sensitivity and specificity in the detection of amblyopia risk factors. One hundred and fifty-five children ranging in age from 4 to 7 years who attended our pediatric ophthalmology clinic were recruited in our study. The Bland-Altman analysis showed moderate agreement between the Spot and cycloplegic retinoscopy, especially for J0 and J45. Spot showed high sensitivity and specificity in detecting amblyopia risk factors based on the criteria from AAPOS 2013 guidelines. The performance could be further improved by optimizing referral criteria based on ROC analysis.

### Comparison between Spot and cycloplegic retinoscopy

In our study, the SE obtained from Spot and cycloplegic retinoscopy was well summarized by a linear regression model. With R^2^ = 0.88, 88 percent of the variance could be explained by this model. Fitting the data with quadratic or cubic model did not improve R^2^ much. From previous study of Plusoptix, the relationship between photoscreener and cycloplegic retinoscopy was best fitted with a non-linear quadratic or cubic model[16]. For Plusoptix A09, even with cubic fitting, the highest R^2^ reached was only 0.73.

Although there was a good linear relationship between the measurements from Spot and retinoscopy, the intercept was not zero. With an intercept of -0.49 D, Spot tended to underestimate hyperopia and overestimate myopia. This tendency was also found in other photscreeners such as Plusoptix. Moghaddam and Dahlmann-Noor’s studies reported a difference of 0.16D and 0.70D between Plusoptix and cycloplegic retinoscopy[17, 18]. We attribute this tendency to the action of accommodation, which was not fully controlled when performed at a distance of one meter away from the children without cycloplegia. For example, if children with myopia less than -1.0 D, emmetropia or hyperopia accommodate exactly onto the target, Spot will report SE values as -1.0 D artifically. In pre-school and school age children, it has been reported that the variability of the accommodation during photorefraction is quite large with some accommodate up to 4.0 D[19]. Several factors, such as the attention of the children, the accommodative stimulus of the environment surrounding the camera, and accommodative lags in hyperopic children, may contribute to this large variability. This may partially explain the wide 95% limits of agreement found in this study.

### Sensitivity, specificity, and the choice of criteria in detecting amblyopia risk factors

With acknowledging of the existing limitations of Spot, this study aimed at improving the effectiveness of the screening by optimizing the referral criteria. The screening process requires the appropriate balance of sensitivity and specificity. High specificity produces adequate positive predictive value for screening. However, since the overall prevalence of amblyopia in the population is low, it is more important to achieve high specificity. An excess of false positive referrals would lead to undesired stress and anxiety to the patient’s family and would also generate unnecessary extra health care cost. Since photoscreeners tend to overestimate myopia and underestimate hyperopia, one should not simply use the criteria meant for identification of amblyopia risk factors defined by cycloplegic refraction to define non-cycloplegic screening referral criteria. To achieve the highest overall effectiveness in detecting amblyopia risk factors, the criteria for specific photoscreener need to be modified based on data comparing the non-cycloplegic screening results to cycloplegic refraction. For example, Garry et al. Reported a sensitivity of 89% and a specificity of 71% of Spot based on the original manufacturer’s criteria (v1.0.3), and a sensitivity of 85% and a specificity of 88% after applying the updated referral criteria (v1.1.51)[10]. The updated criteria (v2.0.16) were also evaluated by Peterseim, who reported a sensitivity of 87.7% and a specificity of 75.9%[12]. More importantly, the criteria should be optimized for specific population due to variations in demographic information. In our study, with optimized criteria at -2.0 D for myopia, the sensitivity and specificity in our study was 94.79% and 85% respectively. With optimized hyperopia criteria at +2.375 D, instead of the +3.5D recommended by AAPOS, the sensitivity increased from 79.66% to 98.31% with minimal sacrifice of specificity. For astigmatism and anisometropia, the optimized criteria were 1.25D and 1.125D respectively. Our findings supported the notion that optimizing the referral criteria for Spot is essential to its success as a screening tool for amblyopia risk factors.

### Comparison of photoscreeners in detecting amblyopia risk factors

A photoscreener is mainly used for the detection of amblyopia risk factors. In our study, Spot had a sensitivity of 94.79% and specificity of 85% for the detection of amblyopia risk factors. Armitage studied the performance of Plusoptix S12 in detecting amblyopia risk factors. The sensitivity and specificity was 91% and 78% respectively [20]. Matta et al. reported that, for Plusoptix S04, the sensitivity was 99% and specificity was 82%[21]. Recently, Yan et al. studied the performance of Plusoptix A09 in detection of amblyopia risk factors in Chinese children who attended the eye clinic, the sensitivity and specificity was 84.7% and 63.2% respectively[16]. It seemed that Spot performed better than Plusoptix S12, Plusoptix A09, and in line with Plusoptix S04, in detection amblyopia risk factors.

### Spot in detecting strabismus

Only manifest strabismus was considered in this study since AAPOS 2013 guidelines does not include intermittent exotropia [13]. After optimized criteria, the sensitivity and specificity for the detection of manifest strabismus were 77.55% and 88.18% respectively. During the measurement with the Spot, we noticed that the results of eye deviation could be affected by head posture. Spot could mistakenly report a normal subject as strabismic, if the subject happened to have a head tilt at the moment when the picture was taken. There should therefore be a method of maintaining head posture, especially in children. Another limitation of this study was that it used clinic patients that had high prevalence of amblyopia risk factors as opposed to community, population, or school based samples of children.

## Conclusion

The refractive values measured from Spot photoscreener showed a moderate agreement of the result from cycloplegic retinoscopy. The performance of Spot in detecting individual amblyopia risk factors was satisfactory, although could be further improved by optimizing criteria based on ROC curves. Those finding suggested that Spot could be a very useful tool for large-scale population screening in Chinese population.
